# An essential role of the reversible electron-bifurcating hydrogenase Hnd for ethanol oxidation in *Solidesulfovibrio fructosivorans*

**DOI:** 10.3389/fmicb.2023.1139276

**Published:** 2023-03-27

**Authors:** Arlette Kpebe, Chloé Guendon, Natalie Payne, Julien Ros, Manel Khelil Berbar, Régine Lebrun, Carole Baffert, Laetitia Shintu, Myriam Brugna

**Affiliations:** ^1^CNRS, Aix-Marseille Univ, BIP, Marseille, France; ^2^CNRS, Aix-Marseille Univ, Centrale Marseille, ISM2, Marseille, France; ^3^CNRS, Aix-Marseille Univ, Plate-forme Protéomique de l’IMM, FR 3479, Marseille Protéomique (MaP), Marseille, France

**Keywords:** hydrogenase, Hnd, *Desulfovibrio*, *Solidesulfovibrio*, ethanol, alcohol dehydrogenase, aldehyde ferredoxin oxidoreductase, flavin-based electron bifurcation

## Abstract

The tetrameric cytoplasmic FeFe hydrogenase Hnd from *Solidesulfovibrio fructosivorans* (formely *Desulfovibrio fructosovorans*) catalyses H_2_ oxidation and couples the exergonic reduction of NAD^+^ to the endergonic reduction of a ferredoxin by using a flavin-based electron-bifurcating mechanism. Regarding its implication in the bacterial physiology, we previously showed that Hnd, which is non-essential when bacteria grow fermentatively on pyruvate, is involved in ethanol metabolism. Under these conditions, it consumes H_2_ to produce reducing equivalents for ethanol production as a fermentative product. In this study, the approach implemented was to compare the two *S. fructosivorans* WT and the *hndD* deletion mutant strains when grown on ethanol as the sole carbon and energy source. Based on the determination of bacterial growth, metabolite consumption and production, gene expression followed by RT-q-PCR, and Hnd protein level followed by mass spectrometry, our results confirm the role of Hnd hydrogenase in the ethanol metabolism and furthermore uncover for the first time an essential function for a *Desulfovibrio* hydrogenase. Hnd is unequivocally required for *S. fructosivorans* growth on ethanol, and we propose that it produces H_2_ from NADH and reduced ferredoxin generated by an alcohol dehydrogenase and an aldehyde ferredoxin oxidoreductase catalyzing the conversion of ethanol into acetate. The produced H_2_ could then be recycled and used for sulfate reduction. Hnd is thus a reversible hydrogenase that operates in H_2_-consumption by an electron-bifurcating mechanism during pyruvate fermentation and in H_2_-production by an electron-confurcating mechanism when the bacterium uses ethanol as electron donor.

## 1. Introduction

*Desulfovibrio* are metabolically versatile sulfate reducing bacteria able to oxidize a wide range of electron donors, such as lactate, pyruvate, or formate in the presence of sulfate but also to ferment certain substrates such as pyruvate. Hydrogen plays a central role in the energy metabolism of these bacteria, since they can use H_2_ as an energy source in the presence of sulfate, or H_2_ is produced during fermentation to dissipate excess reducing power (Fauque et al., 1988; Thauer et al., 2007; Rabus et al., 2013). Moreover, during growth on some substrates, H_2_ is also produced but immediately re-consumed allowing the respiration of sulfate. Several models describing H_2_ metabolism in *Desulfovibrio*, including the H_2_ recycling model, have been proposed over the past 40 years (Odom and Peck, 1981; Lupton et al., 1984; Noguera et al., 1998; Keller and Wall, 2011; Rabus et al., 2013; Sim et al., 2013). Hydrogenases, the key enzymes of this metabolism, catalyze the conversion between H_2_ and protons and electrons. These enzymes, which are functionally and structurally diverse, and widespread in prokaryotes, are classified into two main groups according to the metal content of their active site: FeFe-hydrogenases and NiFe-hydrogenases.

*Solidesulfovibrio fructosivorans* (formerly *Desulfovibrio fructosovorans*, Waite et al., 2020), our model organism, contains no less than six gene clusters encoding different hydrogenases: one membrane-bound of Ech-type, two periplasmic and three cytoplasmic hydrogenases, among which the [FeFe]-hydrogenase Hnd (Baffert et al., 2019). Marker-exchange mutagenesis was performed to determine the role of hydrogenases in the energy metabolism of the bacterium. However, the multiplicity and diversity of these enzymes makes their precise role difficult to establish probably due to compensation processes as the result of deletion of genes encoding one, two, or three hydrogenases (Rousset et al., 1991; Malki et al., 1997; Casalot et al., 2002a,b).

Hnd, a tetrameric enzyme, belongs to the A3 group of the Greening’s FeFe-hydrogenase classification (Greening et al., 2016) which includes hydrogenases that bifurcate electrons. Flavin-based electron bifurcation (FBEB) is a recently discovered mechanism of energy coupling, widely distributed mainly in anaerobic prokaryotes, that couples an endergonic redox reaction to an exergonic redox reaction (for reviews, see; Buckel and Thauer, 2018a,b). Multimeric bifurcating FeFe-hydrogenases couple H_2_ oxidation to the reduction of NAD^+^ and a ferredoxin. Some of them have been shown to perform the reverse reaction and confurcate electrons to produce H_2_ (Schut and Adams, 2009; Schuchmann and Müller, 2012; Wang et al., 2013; Zheng et al., 2014; Kpebe et al., 2018). Contrary to what was previously thought, Hnd reduces NAD^+^ rather than NADP^+^ and a ferredoxin from H_2_ and it retains activity even when purified aerobically unlike other electron-bifurcating hydrogenases (Kpebe et al., 2018; Jacq-Bailly et al., 2020). It contains, in addition to the hydrogenase catalytic subunit (HndD) which is closely related to the CpI hydrogenase from *Clostridium pasteurianum*, a [2Fe-2S] subunit (HndA), a subunit homologous to the NuoF flavin subunit of complex I (HndC), and a fourth subunit (HndB) which probably does not contain any redox center (Malki et al., 1995; Kpebe et al., 2018). The recent structures obtained by cryo-EM of trimeric hydrogenases of this class has allowed to propose a mechanism of electron bifurcation for these hydrogenases (Furlan et al., 2022; Katsyv et al., 2023).

We have recently demonstrated, by comparing the WT strain and the SM4 mutant strain lacking Hnd (Δ*hndD*) (Malki et al., 1997) that this hydrogenase is involved in ethanol metabolism when *S. fructosivorans* grows fermentatively with pyruvate as energy source (Payne et al., 2022, 2023). Whereas under this condition, Hnd is not essential for bacterial growth, the deletion of *hndD* leads to a dramatic decrease in the expression of two genes involved in the conversion of acetate into ethanol: *adh* (DesfrDRAFT_3929) encoding an iron-containing alcohol dehydrogenase (Adh) and *aor* (DesfrDRAFT_2487) encoding an aldehyde ferredoxin oxidoreductase (Aor). The Δ*hndD* mutant strain is, under these conditions, affected in the production of H_2_ and ethanol. We proposed a model in which the oxidation of H_2_ by Hnd would allow, thanks to its electron bifurcation mechanism, the production of reducing power in the form of NADH and reduced ferredoxin necessary for the production of ethanol as a fermentation product by the two enzymes Adh3929 and Aor2487 (Payne et al., 2022). In addition, our NMR-based metabolomic analysis highlighted that, under these growth conditions, the metabolic reprogramming induced by the deletion of *hndD* leads to the upregulation of several NADP-dependent pathways, including succinate production and branched-chain amino acid biosynthesis pathways (Payne et al., 2023).

To further understand the precise role of Hnd and since this hydrogenase is involved in ethanol metabolism, the approach implemented in this study was to compare the two *S. fructosivorans* WT and SM4 Δ*hndD* strains when grown on ethanol as the sole carbon and energy source. Our results confirm the role of this electron-bifurcating hydrogenase in ethanol metabolism and furthermore uncover for the first time an essential function for a *Desulfovibrio* hydrogenase.

## 2. Materials and methods

### 2.1. Bacterial strains and growth conditions

The WT strain of *S. fructosivorans* JJ (Ollivier et al., 1988), the SM4 strain lacking Hnd, deleted of *hndD* encoding the hydrogenase catalytic subunit (Δ*hndD*, Cm^R^) (Malki et al., 1997), and the complementation strain, containing the entire *hnd* operon on the plasmid pBGhnd6 (strain SM4/pBGhnd6, Cm^R^, and Gm^R^) (Kpebe et al., 2018) were used in this study. The ADH-CG1 mutant strain of *S. fructosivorans*, lacking Adh3929, obtained in this study was also used.

Strains were grown anaerobically at 37°C in different media, containing pyruvate as carbon and electron donor and sulfate as electron acceptor (PS2 and PS20 media described in Payne et al., 2022) or containing ethanol instead of pyruvate as carbon and electron donor, in the presence of 20 mM sulfate (ES20 medium) or in the absence of sulfate (ES0 medium). Ethanol was used at the concentration of 40 mM for the WT and complementation strains, whereas for the SM4 strain the concentration was 60 mM because of the addition of the antibiotics thiamphenicol that is dissolved in ethanol. Thiamphenicol (34 μg/mL) was added to the medium for the growth of SM4 and ADH-CG1 strains and gentamicin (20 μg/mL) was added for the growth of SM4/pBGhnd6 strain. The media were inoculated with 5–10% of fresh cultures grown in PS2 medium. Growth on molecular hydrogen was also tested in the same medium (but without pyruvate or ethanol) in the presence of 30 mM sulfate, 10 mM acetate and 1 bar pressure of H_2_, in 90 mL of anaerobic medium in 100 mL serum bottles. The medium was bubbled 15 min with H_2_ before the inoculation with cells_._

### 2.2. Construction of the *adh* mutant (ADH-CG1 strain)

The strain, called ADH-CG1, lacking the *adh* gene (accession WP_005996817, locus tag DESFRDRAFT_RS19365, old locus tag DesfrDRAFT_3929) was constructed for this study. In order to replace the entire *adh* gene by the chloramphenicol *Cm^R^* cassette on the chromosome, a 1,087 bp and a 1,086 bp PCR fragments corresponding to the upstream and downstream region of *adh* gene were cloned in the pNot19Cm plasmid (Fiévet et al., 2011) surrounding the Cm^R^ gene. Upstream and downstream region of *adh* gene were amplified with the following primers: ADH3929-del-amont-slic-pnot-F (5′CTCTAGAGGCGCGCCACTAGTCAGAAAGCGAGGGACTACTTC3′), ADH3929-del-amont-slic-cm-R(5′AACAGGGAAGTGAGAACTAGGGTGCCAACCTCACTTGGGT3′), ADH3929-del-aval-slic-F(5′AAATTCGCAATTGAGATCTATACGGGACCAACGTGTCAAA3′), ADH3929-del-aval-slic-R (5′GACCATGATTACGCCAAGCTTCTCATGAATTCGACTCCCTC3′). The *Cm^R^* gene (1,413 bp) was amplified with the following primers: Cm slic-del-ADH3929-F(5′ACCCAAGTGAGGTTGGCACCCTAGTTCTCACTTCCCTGTT3′), Cm slic-del-ADH3929-R (5′TTTGACACGTTGGTCCCGTATAGATCTCAATTGCGAATTT3′). The three fragments were cloned in the SpeI/ HindIII digested pNot19Cm plasmid using the NEBuilder HiFi DNA assembly mix from NEB according the instruction. The pNot19Cm is a derivative pUC19 vectors and do not replicate in sulfate-reducing bacteria, it is used as a suicide plasmid in *S. fructosivorans* to direct the marker exchange mutagenesis on the chromosome. The resulting plasmid pNot-Cm-adh3929 was introduced in the WT strain of *S. fructosivorans* using electrotransformation (Malki et al., 1997). The genotype of the chloramphenicol/thiamphenicol resistant strain was analyzed by PCR and DNA sequencing to confirm the deletion of *adh* gene.

### 2.3. Metabolite production and consumption

Strains were grown in 100 mL serum bottles containing 90 mL of medium. Each culture was done in triplicate. H_2_ accumulation was followed as described previously using a gas chromatography system (Agilent 7820A, GS-Carbonplot 115–3,133 column) (Payne et al., 2022). The H_2_ accumulation is expressed as micromoles of H_2_ accumulated in the headspace. Ethanol consumption and acetate production were followed by HPLC as previously described (Payne et al., 2022). The analysis was performed on the same *S. fructosivorans* cultures as those used for the growth curves and as those used to measure H_2_ production and sulfate consumption. The consumption of sulfate in the growth medium was followed using ionic chromatography. At different growth times, 1 mL of culture samples were collected, centrifuged 5 min at 14,100 × g to remove cells and stored at −20°C until used. Before injection, samples were diluted in 1% isopropanol up to 7.5 mL and 0.22 μm filter-sterilized. Samples were analyzed using a Metrohm (Herisau Switzerland) 925 ECO ion chromatograph equipped with a conductivity detector, Metrohm Suppressor Module (MSM), 250 μL injection loop with peristaltic pump, Metrohm high pressure pump with purge valve and 863 compact autosampler. The detection of SO_4_^2−^ anions was investigated, at room temperature, in suppressor mode using the separation column “Metrosep A Supp 4” (250 mm × 4.0 mm) with a Metrohm IC precolumn cartridge PRP-2 (Metrosep RP2 Guard/3.5) and eluent 1.7 mM NaHCO_3_/1.8 mM Na_2_CO_3_, 20% acetone. The eluent flow-rate was 1 mL/min. The calibration of the instrument was done by injecting standard solutions of sulfate. The instrument was operated with the MagIC Net Basic software, that controls and monitors the instrument, evaluates the recorded data and manages it in a database.

### 2.4. Protein extract preparation

Preparation of protein extracts was done as follows: *S. fructosivorans* cells, grown in 250 mL bottles containing 200 mL of medium were harvested at 3 days after the end of the exponential phase and resuspended in 4 mL of 20 mM Tris–HCl pH 7.5 supplemented with 2 mM sodium dithionite, protease inhibitor cocktail (SIGMAFAST™ tablet), Dnase I (Roche) and Lysozyme (Sigma-Aldrich). The cells were broken in an anaerobic glovebox (Jacomex) using a sonicator (Hielscher) with 15 cycles of 30 s. Non-disrupted cells and cellular debris were removed by a centrifugation at 20,000× *g* for 20 min. Total cell extract was ultracentrifuged at 200,000× *g* for 45 min at 4°C, then the soluble extract was recovered. For hydrogenase enzymatic assay, the same protocol is carried out except that cells were harvested at the beginning of the stationary phase, the pellet was resuspended in 100 mM Tris–HCl pH 8 buffer and that total protein extract was used in the tests. Proteins were quantified using the Bio-Rad protein assay based on Bradford dye binding method using bovine serum albumin as standard. The same soluble protein extracts were used for ethanol dehydrogenase enzymatic assays, SDS-PAGE, Western immunoblot, and mass spectrometry analysis.

### 2.5. SDS-PAGE and Western blot

Separation of proteins by 10% SDS-PAGE and Western immunoblot were described previously (Payne et al., 2022). Two identical 10% SDS-PAGE were performed in parallel with 15 μg of soluble protein extract. After migration, the first one was stained with a Coomassie blue solution and the second one was used for western blotting experiment. The primary anti-HndD antibody was used to detect HndD protein on the nitrocellulose membrane.

### 2.6. Cloning, production, and purification of proteins

Recombinant strep-tagged Hnd hydrogenase and FdxB ferredoxin, both from *S. fructosivorans*, were produced and purified as described previously (Kpebe et al., 2018). A concentration of 1.46 mg/mL for Hnd was determined using the Bio-Rad protein assay based on Bradford dye binding method using bovine serum albumin as standard. The concentration of the mature FdxB in oxidized form was estimated to be 233 μmol/L by spectrophotometry at 410 nm, corresponding to the absorption peak of iron sulfur clusters, using an absorption coefficient value of 24,000 M^−1^ cm^−1^.

The same strategy for *fdx*B cloning was used for *pfor* gene cloning. The *pfor* gene encoding the pyruvate:ferredoxin oxidoreductase (accession WP_006919792.1, locus tag DESFRDRAFT_RS00590, old locus tag DesfrDRAFT_0121) was amplified using the following primers: Oligo1-PFOR-df-SLIC-F (5′GTTTATCTGTTACCCCGTAGGATCCATGGCCAAGAAGATGAAAAGC3′), Oligo2c-PFOR-strep-df-SLIC-R(5′ACTACCACCACCACTACCCCCGGGTTTTCCGGAGCGGCCTTCGT3′). The 3,687 bp PFOR PCR fragment was digested with *SmaI* and *BamHI* NEB enzymes and cloned into the *SmaI-BamHI* linearized pthl-Fd-LL-C-Tag plasmid (Gauquelin et al., 2018). The resulting pthl-PFOR-LL-C-Tag plasmid was checked by sequencing and introduced in the *E. coli* MG1655 *∆iscR::kan* strain by electroporation. The resulting strain was then used to produce recombinant strep-tagged PFOR. Strain was grown aerobically at 37°C during 16 h in LB medium supplemented with 0.5% glycerol, 40 mM sodium fumarate, 0.1 g/l Fe(III) citrate, 40 mM MOPS, 50 μg/mL kanamycin and 100 μg/mL ampicillin. Cells pellet was washed and then resuspended in buffer W (Tris–HCl 100 mM pH 8.0, NaCl 150 mM) supplemented with SIGMAFAST protease Inhibitor Cocktail tablet (SIGMA) and DNaseI (Roche). All purification steps were done anaerobically in a glove box under nitrogen atmosphere (Jacomex). Cells were lysed by sonication (20 cycles of 30 s). After ultracentrifugation (45 min at 200,000 × g at 4°C), the supernatant was loaded on a 5 mL StrepTactin superflow (IBA) affinity column and purification was done according IBA instruction. PFOR was eluted with 2.5 mM desthiobiotin in buffer W, dialyzed and concentrated with a 30 kDa molecular mass cut-off Vivaspin 20 (Sartorius). The purity of the PFOR (133.4 kDa) was checked on a 10% SDS-PAGE (Supplementary Figure S1). The catalytic activity of recombinant PFOR was measured in an anaerobic quartz cuvette, at 30°C, under N_2_ atmosphere, in 1 mL reaction mixture containing 1 mM MgCl_2_, 2 mM thiamine pyrophosphate, 0.32 mM dithioerythritol, 10 mM sodium pyruvate, 0.1 mM Coenzyme A, 20 μM ferredoxin FdxB in 100 mM Phosphate buffer pH7. The reaction was started by the addition of PFOR in the cuvette and absorbance at 410 nm was followed (reduction of FdxB). An activity of 0.045 U/mg was determined.

### 2.7. Enzymatic assays

Hydrogenase activity, both H_2_-oxidation and H_2_-evolution, using the methylviologen as electron acceptor/donor, in total cell extracts was measured as described previously (Payne et al., 2022). One unit of hydrogenase activity corresponds to the uptake of 1 μmol of H_2_/min and the production of 1 μmol of H_2_/min, respectively.

Electron bifurcation activity of Hnd was determined as described in Kpebe et al. (2018). NADH- and Fd-dependent H_2_-production activity of Hnd (electron confurcation assay) was assayed using gas chromatography (GC). A 800 μL mixture containing 100 mM Tris–HCl pH 8.0, 4 μM of FMN, 4 μM of FAD, 15 mM sodium pyruvate, 25 μM Coenzyme A, 1 mM MgCl_2_, 2 mM thiamine pyrophosphate, 0.32 mM dithioerythritol, 2.4 mM NADH, 73 μM ferredoxin FdxB, 36 μg of PFOR was prepared in a glove-box (Jacomex) and placed in a 8 mL vial closed by a septum. The reaction mixture was let react at room temperature for 40 min to allow the maximum reduction of FdxB by the PFOR. The confurcation reaction was started by the addition of 14.6 or 43.8 μg of Hnd. Hundred microliter of headspace gas were removed periodically using a gastight syringe and injected into a gas chromatography system (Agilent 7,820) equipped with a thermal conductivity detector and a HP-plot Molesieve capillary column (30 m, 0.53 mm, 25 μm), using argon as the carrier gas, at a flow rate of 4.2 mL/min, an oven temperature of 30°C and a detector temperature of 150°C. H_2_ was quantified according to a standard calibration curve. H_2_ production rate was expressed as μmol of H_2_ produced. The H_2_ signal appears for a retention time of 2.1 min while O_2_ retention time is 2.5 min. Thus we can discriminate between the signals of these two gases. Electron bifurcation and confurcation activities were measured using the same samples of Hnd and ferredoxin FdxB proteins.

Ethanol dehydrogenase activity was determined in soluble cell extract according to Ramos et al. (2015) except that the assays were not performed in an anaerobic glove box but the activity was measured using anaerobic spectrophotometric cuvette. As a control, NAD^+^ reduction was followed without ethanol in the reaction mixture in order to determine the NAD^+^ reduction independent of ethanol dehydrogenase activity, which reduction was systematically deduced from the value measured in the reaction with ethanol.

### 2.8. Real-time quantitative PCR

10^10^ cells were pelleted. Total RNA was extracted using the *Maxwell*^®^ Instruments from PROMEGA. This automated nucleic acid purification platform can perform 16 samples simultaneously. The cells pellet was resuspended in the homogenizing solution (*Maxwell Promega kit*). The resuspended cells were mixed with 0.1 mm glass beads followed by two 45 s steps of 6.5 m/s in the QUICKPREP mode of the MP Biomedicals™ Instrument FastPrep-24™ 5G. The total RNA extraction was then carried out according to the supplier’s protocol. An extra step, with *TURBO DNA free™ kit (Life technology)* was done to remove genomic DNA contamination of the total RNA. RNA concentration was determined with a NanoDrop 2000 spectrophotometer and purity was checked with the automated platform of Agilent 4200 TapeStation system. Reverse transcription was performed on 1 μg of total RNA by using the superscript VI reverse transcriptase and random primers (Invitrogen). The protocol and primers used were previously described by Payne et al. (2022).

### 2.9. Relative quantitative proteomic data analysis

The protein-containing SDS-PAGE stacking bands containing 60 μg of extracted proteins from cells of *S. fructosivorans* WT and SM4/pBGhnd6 strains cultured in PS2 and ES20 conditions and collected at stationary phase of growth, were treated in biological triplicates for proteomic analysis as previously described in Gérard et al. (2022) with slight modifications as follows. Eight hundred nanograms of peptides were injected for LC-MSMS analysis on nano liquid chromatography Ultimate 3,000 (Thermo Scientific) coupled to a Q-Exactive Plus mass spectrometer (Thermo Scientific). The spectra were processed by Proteome Discoverer software (ThermoFisher, version: 2.4.1.15) using the Sequest HT algorithm with the search following settings: *Solidesulfovibrio_fructosivorans* database (TxID 596151) extracted from Uniprot (4,161 sequence entries); trypsin enzyme (maximum 2 missed cleavages); fixed modification: carbamidomethyl (Cys); variable modification: oxidation (Met); mass values specific for monoisotopic; precursor mass tolerance: ± 10 ppm; fragment mass tolerance: ± 0.02 Da; spectrum files were recalibrated. Peptide validation was based on target/decoy strategy based on *q*-value, maximum Delta Cn 0.05 and Strict Target False Discovery Rate at 0.01. Proteins were identified if minimum 2 unique peptide sequences more than 6 amino acids passed the high confidence filter. Relative quantification of Adh3929 (E1K229), Hnd (HndA, E1JRZ9; HndB, E1HS00; HndC, E1JS01; HndD, E1JS02) and Aor2487 (E1JXY8) between the different conditions was established based on the number of Peptide Spectral Matched (PSM) of a protein, normalized by the total number of PSM in each sample, and averaged by the biological triplicate. Comparison was performed against the proteins from the WT strain grown in PS2 condition, fixed at 100%.

## 3. Results

### 3.1. SM4 mutant phenotype in ethanol medium

**Figure 1:**
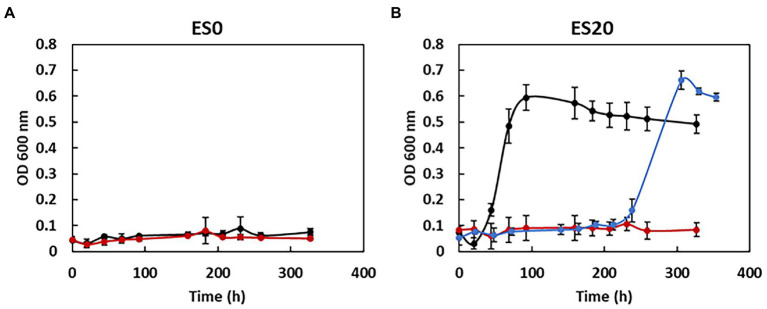
Growth curves of *Solidesulfovibrio fructosivorans* WT, SM4, and SM4/pBGhnd6 strains. Bacteria were grown in 100 mL serum bottles containing 90 mL of ES0 **(A)** and ES20 **(B)** media supplemented or not with 20 mM sulfate, respectively, and 40 or 60 mM ethanol as electron donor for the WT and the complementation strains, and the mutant strain, respectively. WT strain, black curves; SM4 strain, red curves; SM4/pBGhnd6 strain, blue curve. Data represent the averages of the results of three or four replicate growths. Error bars correspond to standard deviations.

Both *S. fructosivorans* strains, the WT strain and the SM4 mutant strain deleted of *hndD* encoding the catalytic subunit of the Hnd hydrogenase, were grown on media that contain only ethanol as a carbon and energy source in the presence of 20 mM sulfate (ES20 medium) and in the absence of sulfate (ES0 medium). Growth was monitored over time by measuring the OD of cultures at 600 nm (Figure 1). As expected, in the presence of sulfate, on ES20 medium, the WT strain was able to oxidize ethanol for growth. The culture reached the stationary phase of growth after 90 h and the doubling time was 12.1 ± 1.9 h. In contrast, when sulfate was absent, the WT strain did not grow. Indeed, the bacterium is not able to ferment ethanol (Ollivier et al., 1988). The SM4 mutant strain is unable to grow on ethanol in both media, either in the presence or in absence of sulfate. These findings demonstrate that the deletion of *hndD* makes it impossible for this mutant strain to grow on ethanol even in the presence of sulfate, therefore the Hnd hydrogenase is essential for the growth of *S. fructosivorans* on ethanol. It should be noted that the SM4 mutant strain grows well under other growth conditions, on pyruvate, lactate and fructose (Malki et al., 1997; Payne et al., 2022, 2023). The deletion of *hndD* does not affect the growth of the bacterium on H_2_/sulfate medium (Supplementary Figure S2) which confirms the result obtained by Malki et al. (1997) who showed that the growth of a mutant lacking Hnd (Δ*hndC*) is not affected compared to the WT strain. Hnd is not essential for bacterial growth on H_2_/sulfate.

To confirm that the observed phenotype is indeed caused by the *hndD* deletion, the SM4 mutant strain carrying a plasmid containing the *hnd* operon (strain SM4/pBGhnd6, Kpebe et al., 2018) was cultivated on the two media ES0 and ES20. We observed that this strain, like the WT strain, can grow on ethanol in the presence of sulfate (Figure 1) but not in its absence (not shown). While the highest optical density at 600 nm is similar for both strains, the doubling time of the SM4/pBGhnd6 complementation strain is 4 times higher than that of the WT strain (47.9 ± 4.1 h). These results indicate that the expression of *hnd* from the pBGhnd6 plasmid restores the growth in a medium containing ethanol as the only source of carbon and energy although the complementation strain does not grow as fast as the WT strain. These results confirm that Hnd is essential for ethanol oxidation in *S. fructosivorans*. It is of note that, for some unknown reasons, the growth curve of the complemented strain shows a significant lag, the maximal OD600 is reached at 92 h for the WT strain while it is only reached at 306 h for the complemented strain. Pre-cultures of the strains were done on pyruvate medium and it seems that the complementation strain needs an adaptation time when transferred to ethanol medium. A lag phase of 200–250 h was also observed for a hydrogenase mutant of *Desulfovibrio vulgaris* Hildenborough (DvH) when the strain is transferred from a lactate medium to an ethanol medium (Haveman et al., 2003).

### 3.2. Metabolite production and consumption

**Figure 2:**
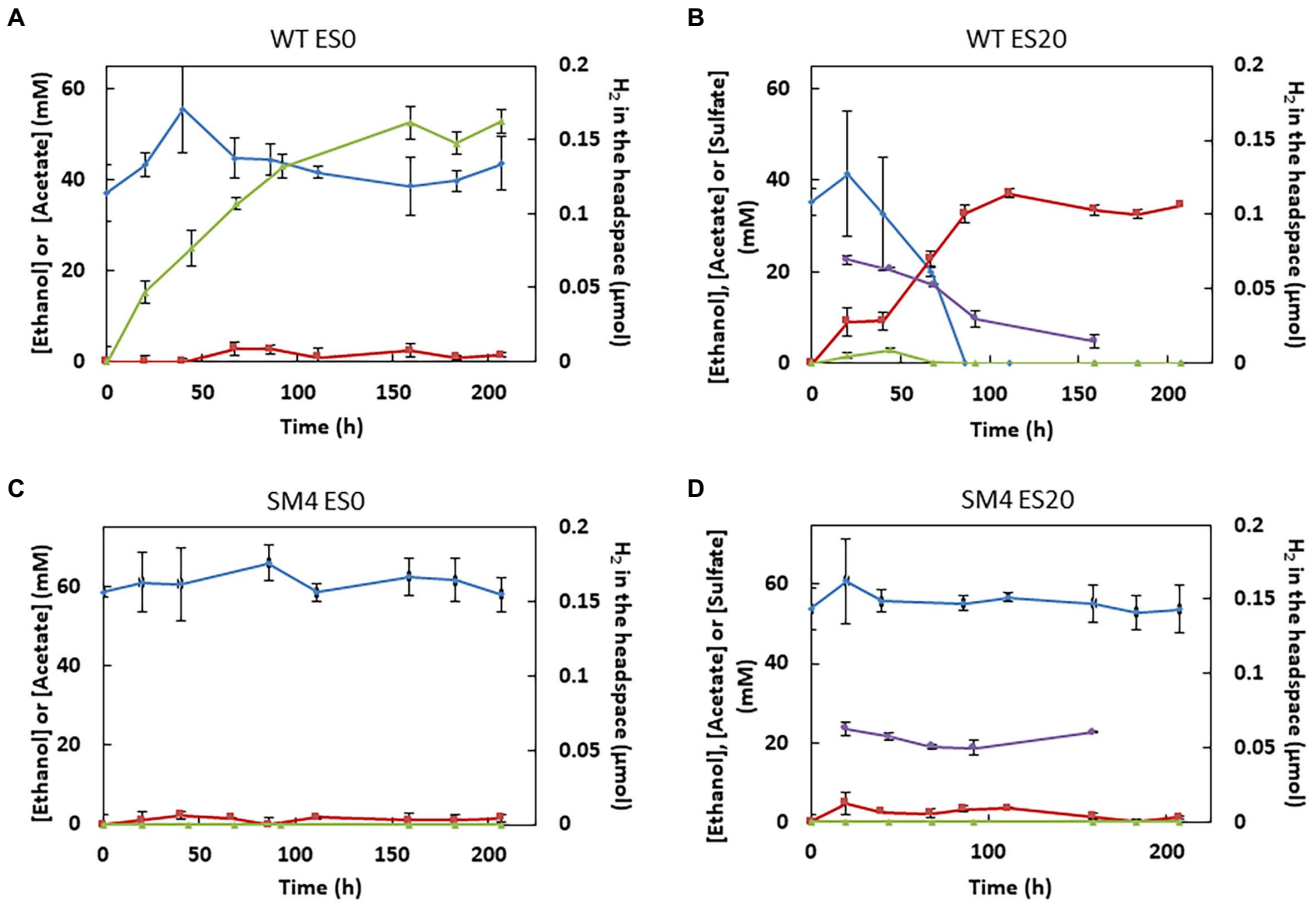
Acetate, ethanol, sulfate, and H_2_ quantification. Acetate (red curves), ethanol (blue curves), and sulfate (purple curves) in the growth medium and H_2_ in the headspace (green curves) were quantified by HPLC, ionic chromatography or GC during the growth of the *S. fructosivorans* WT strain **(A,B)** or the SM4 mutant strain **(C,D)** in the ES0 medium **(A,C)** or the ES20 medium **(B,D)**. Analyses were performed on the same *S. fructosivorans* cultures as those used for the growth curves. Data represent the averages of the results of three replicate growths. Error bars correspond to standard deviations.

In order to determine the effect of *hndD* deletion on the production of major metabolites, acetate and H_2_ production as well as ethanol and sulfate consumption were followed during growth (Figure 2). As expected, ethanol is consumed quite rapidly (in less than 100 h) by the WT strain on ES20 medium whereas in absence of sulfate, ethanol is not consumed at all. Ethanol oxidation is coupled to sulfate reduction. This respiration allows the growth of the bacterium while fermentation is not possible. The mutant strain does not consume ethanol on both ES20 and ES0 media, explaining why it does not grow under these conditions. Acetate is produced and sulfate is consumed only when ethanol is oxidized by the WT strain in ES20 medium.

H_2_ accumulates in the gas phase when the WT strain grows in the absence of sulfate, showing that even though *S. fructosivorans* does not grow under these conditions, the bacteria from the inoculum are still able to survive and produce H_2_. On ES20 medium, on the other hand, there is no accumulation of H_2_ in the headspace although we observe a transient burst of H_2_ at the beginning of the growth. In contrast, the SM4 mutant strain does not produce H_2_ at all.

Taken together, these results lead us to propose that the Hnd hydrogenase catalyzes H_2_ production when *S. fructosivorans* grows with ethanol as the only energy source in the presence of sulfate. It would therefore carry out the confurcation of electrons from NADH and reduced ferredoxin. We check the ability of Hnd to confurcate electrons *in vitro*. We determine an activity of 0.033 ± 0.015 U/mg. Although this activity is low compared to electron bifurcation activity (0.96 U/mg determined with the same protein sample), it shows that Hnd is able to produce H_2_ *in vitro* from reduced FdxB ferredoxin and NADH (Supplementary Figure S3).

### 3.3. Impact of the carbon source and growth condition on Hnd level

**Figure 3:**
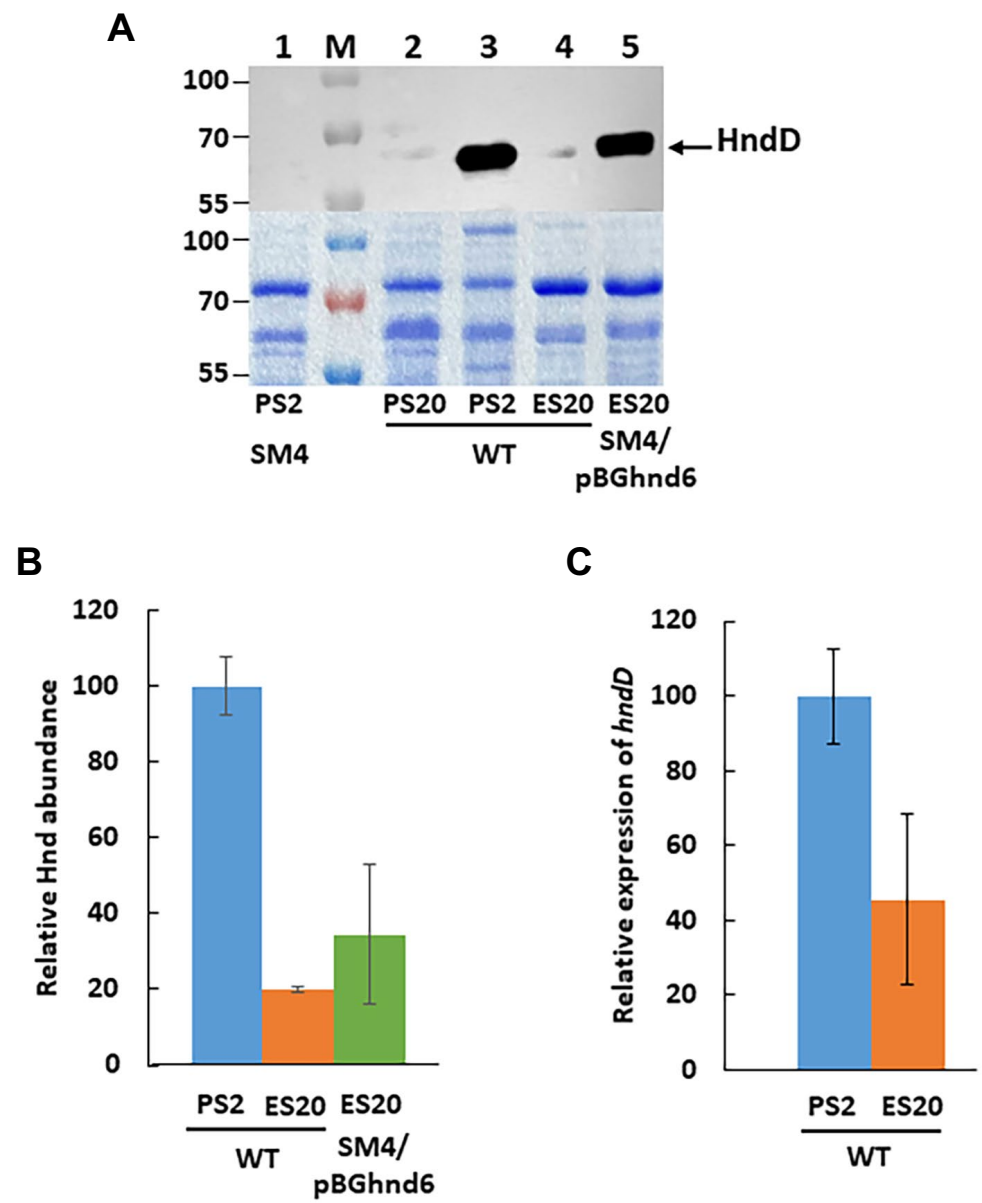
Hnd level in *S. fructosivorans* under pyruvate and ethanol conditions. **(A)** Western blot analysis of HndD production by WT (lanes 2–4), SM4 (lane 1) and SM4/pBGhnd6 (lane 5) cells of *S. fructosivorans* grown with pyruvate (PS2, lanes 1 and 3, or PS20, lane 2) or ethanol (ES20, lanes 4 and 5) as carbon source. Cells were harvested at stationary phase of growth and 15 μg of total proteins from soluble extracts were separated by 10% SDS-PAGE and subjected to western blotting using an antibody raised against HndD. Molecular mass markers (lane M) are indicated in kDa. The SDS-PAGE below the western blot is a loading control. **(B)** Relative Hnd level determined by quantitative proteomics. *S. fructosivorans* WT and SM4/pBGhnd6 strains were grown with pyruvate (PS2) or ethanol (ES20) as carbon source and cells were collected at stationary phase of growth. Data represent the averages of the results of three experiments. Error bars correspond to standard deviations. **(C)** Relative expression of the *hndD* (DesfrDRAFT_0401) gene quantified by RT-qPCR. *S. fructosivorans* WT strain was grown with pyruvate (PS2) or ethanol (ES20) as carbon source and samples were collected at stationary phase of growth. Expression of the gene was normalized to that of the 16S rRNA gene. Data represent the averages of the results of three experiments. Error bars correspond to standard deviations.

Hnd production in the WT strain, followed by Western blot, was compared under pyruvate (fermentation or respiration) and ethanol (respiration) conditions (Figure 3A). As expected, in pyruvate medium, the amount of Hnd is much higher in fermentation (PS2) than in respiration (PS20) (Payne et al., 2022). Surprisingly, Hnd is much less abundant when the bacterium is growing in ethanol medium (ES20) than in PS2 medium, while it is essential for the growth of the bacterium when growing on ethanol as carbon source (Figure 1). To confirm this result, Hnd abundance was measured by quantitative proteomics using spectral counting from soluble extracts of the WT strain (Figure 3B) and an amount of Hnd 5 times lower was observed when bacteria were grown on ethanol medium (ES20) as compared to pyruvate fermentation medium (PS2). The expression of the *hndD* gene (locus tag DesfrDRAFT_0401), which was monitored by RT-qPCR, is more than twofold lower for the WT strain grown in ethanol medium (ES20) than in pyruvate medium (PS2) (Figure 3C). Expression of *hnd* is consistent with the results obtained at the Hnd protein level.

We measured the hydrogenase activity of H_2_-consumption and H_2_-production from total extracts of the WT and the SM4 mutant strains grown on the three media (PS2, PS20, and ES20) (Table 1). As shown previously, Hnd is responsible for the majority of the hydrogenase activity measured in the WT in PS2 medium since it is strongly reduced in the mutant SM4 strain (Payne et al., 2022). In PS20, the decrease in hydrogenase activity as compared to PS2 medium is explained by the drastic decrease in *hndD* expression (Payne et al., 2022). Interestingly, the activity of the WT strain is about 10 times lower on ES20 than on PS2 for H_2_-oxidation and a half for H_2_-production. This sharp drop in both hydrogenase activities under ethanol compared to pyruvate fermentation conditions correlates with the significant decrease in the amount of the Hnd hydrogenase when the bacteria are growing on ethanol (Figures 3A,B).

**Table 1:** Hydrogenase activity in *S. fructosivorans*.

Growth medium		Hydrogenase activity (U.mg^−1^)	
	*S. fructosivorans* strain	H_2_-oxidation	H_2_-production
PS2	WT	28.7 ± 6.0	8.4 ± 2.7
	SM4	0.4 ± 0.1	0.9 ± 0.1
PS20	WT	5.0 ± 1.6	1.6 ± 0.3
	SM4	2.7 ± 0.5	1.3 ± 0.5
ES20	WT	3.3 ± 1.0	4.3 ± 1.4
	SM4/pBGhnd6	6.9 ± 3.0	4.2 ± 1.2

H_2_-production and H_2_-oxidation were measured on the total extract of the WT, the SM4 mutant and the complementation SM4/pBGhnd6 strains grown with pyruvate (PS2 and PS20) or ethanol (ES20) as carbon source. Cells were harvested at the end of exponential phase of growth.

The anti-HndD western blot on the complementation strain shows that, on ES20 ethanol medium, the amount of Hnd is similar to that of the WT strain, indicating that Hnd production is restored from the operon on the pBGhnd6 plasmid (Figure 3A). The same pattern is observed when the abundance of Hnd is determined by quantitative proteomics (Figure 3B). These results correlate with the fact that H_2_ production and oxidation activities of the SM4/pBGhnd6 strain are comparable to those measured for the WT strain (Table 1).

Collectively, these results clearly show that the expression of the *hnd* operon as well as the amount of Hnd hydrogenase are much lower when the bacteria are growing under respiration (PS20 or ES20) than under fermentation (PS2). Unexpectedly, under respiratory condition, the nature of the carbon source, pyruvate or ethanol, has little effect on the expression of the hydrogenase while this enzyme is essential for the growth of the bacteria on ethanol but not on pyruvate (Payne et al., 2022).

### 3.4. Impact of the carbon source and growth condition on Adh and Aor levels

In a previous study, we have demonstrated that the deletion of the *hndD* gene, in the SM4 strain, leads to a drastic drop in the expression of the *adh*3929 gene (locus tag DesfrDRAFT_3929), coding for an alcohol dehydrogenase, and of the *aor*2487 (DesfrDRAFT_2487) coding for an aldehyde ferredoxin oxidoreductase (Payne et al., 2022). We therefore monitored Adh and Aor at the protein as well as the transcriptional levels under different growth conditions.

**Figure 4:**
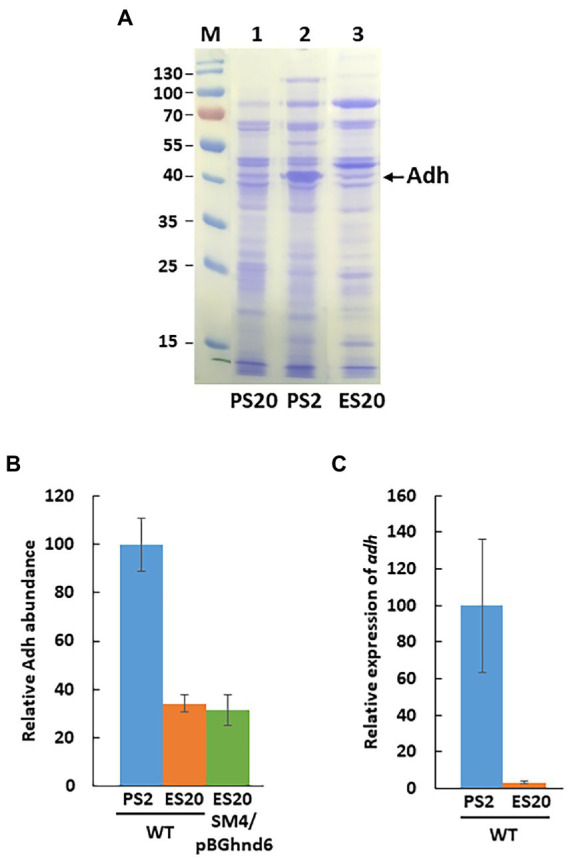
Adh level in *S. fructosivorans* under pyruvate and ethanol conditions. **(A)** Coomassie blue-stained SDS-PAGE of soluble proteins from *S. fructosivorans* WT strain grown with pyruvate (PS20, lane 1 or PS2, lane 2) or ethanol (ES20, lane 3) as carbon source. Cells were harvested at stationary phase of growth. Ten micrograms of soluble proteins were loaded in each lane of a 12% SDS-PAGE. Molecular mass markers (lane M) are indicated in kDa. Arrow indicates bands corresponding to Adh (3929). The theoretical molecular mass of Adh is 41.9 kDa. **(B)** Relative Adh level determined by quantitative proteomics. *S. fructosivorans* WT and SM4/pBGhnd6 strains were grown with pyruvate (PS2) or ethanol (ES20) as carbon source and cells were collected at stationary phase of growth. Data represent the averages of the results of three experiments. Error bars correspond to standard deviations. **(C)** Relative expression of the *adh* (DesfrDRAFT_3929) gene quantified by RT-qPCR. *S. fructosivorans* WT strain was grown with pyruvate (PS2) or ethanol (ES20) as carbon source and samples were collected at stationary phase of growth. Expression of the gene was normalized to that of the 16S rRNA gene. Data represent the averages of the results of three experiments. Error bars correspond to standard deviations.

The amount of Adh3929 is so high in the cell in PS2 that it can be visualized directly on SDS-PAGE of cell extracts (Payne et al., 2022). Following Adh during bacterial growth shows that the enzyme accumulates in the cells (Supplementary Figure S4). Comparison of the amount of Adh in the stationary phase reveals that it is much more abundant in *S. fructosivorans* WT cells grown on PS2 than under respiration in PS20 medium as already shown (Payne et al., 2022) but also in ES20 medium (Figure 4A). Abundance of Adh was also measured by quantitative proteomics and confirms that the quantity of the enzyme is 3 times lower under ethanol respiration (ES20) than in pyruvate fermentation (PS2) for the WT strain (Figure 4B).

The ethanol dehydrogenase specific activity was determined on soluble extracts of the WT and the SM4 mutant strains (Table 2). The SM4 mutant shows no detectable ethanol dehydrogenase activity on pyruvate medium (PS2 and PS20). This is consistent with the fact that in this mutant the expression of *adh* is strongly reduced compared to the WT strain (Payne et al., 2022). More importantly, what was less expected is that in respiration, the ethanol dehydrogenase activity is similar on pyruvate medium (PS20) as on ethanol medium (ES20) for the WT strain. This activity is considerably lower when the bacteria are growing under ethanol respiration (ES20) than on pyruvate fermentation (PS2).

**Table 2:** Ethanol dehydrogenase activity in *S. fructosivorans*.

Growth medium	*S. fructosivorans* strain	Ethanol dehydrogenase activity (mU.mg^−1^)
PS2	WT	375 ± 38
	SM4	N.D.
	SM4/pBGhnd6	4 ± 1
PS20	WT	18 ± 12
	SM4	N.D.
	SM4/pBGhnd6	1 ± 1
ES20	WT	22 ± 15
	SM4/pBGhnd6	51 ± 12

Ethanol dehydrogenase activity was measured on the soluble extract of the WT, the SM4 mutant and the complementation SM4/pBGhnd6 strains grown with pyruvate (PS2 and PS20) or ethanol (ES20) as carbon source. Cells were harvested at the stationary phase of growth. N.D., not detected.

Similarly, the *adh* gene expression, measured by RT-qPCR, is extremely higher on PS2 than on ethanol medium (ES20) (Figure 4C). This result is consistent with the lower amount of Adh enzyme present on ES20 than on PS2 (Figures 4A,B) and also with the ethanol dehydrogenase activity measured under these two conditions (Table 2). The presence of 40 mM of ethanol in the growth medium does not induce the overexpression of the *adh* gene at the transcriptional level.

The ethanol dehydrogenase activity for the complementation strain is very low in pyruvate media (PS2 and PS20) (Table 2) which is consistent with the fact that the complementation is only partial when the SM4/pBGhnd6 strain is growing on pyruvate (Payne et al., 2022). In contrast, when the complementation strain is grown on ethanol medium, expression of *hnd* operon from the plasmid restores Adh (both the amount of the enzyme and the ethanol dehydrogenase activity) suggesting that *adh* expression is restored (Figure 4B and Table 2).

**Figure 5:**
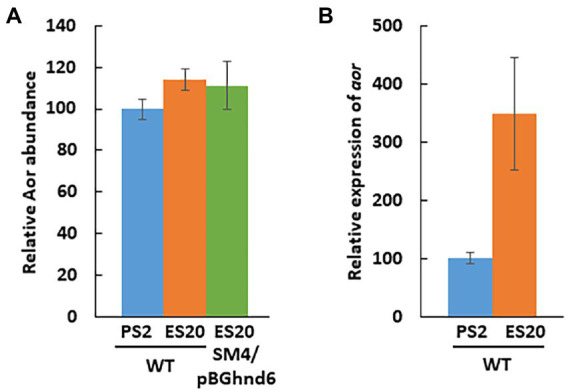
Aor level in *S. fructosivorans* under pyruvate and ethanol conditions. **(A)** Relative Aor level determined by quantitative proteomics. *S. fructosivorans* WT and SM4/pBGhnd6 strains were grown with pyruvate (PS2) or ethanol (ES20) as carbon source and cells were collected at stationary phase of growth. Data represent the averages of the results of three experiments. Error bars correspond to standard deviations. **(B)** Relative expression of the *aor* (DesfrDRAFT_2487) gene quantified by RT-qPCR. *S. fructosivorans* WT strain was grown with pyruvate (PS2) or ethanol (ES20) as carbon source and samples were collected at stationary phase of growth. Expression of the gene was normalized to that of the 16S rRNA gene. Data represent the averages of the results of three experiments. Error bars correspond to standard deviations.

Regarding Aor, quantitative proteomics and RT-qPCR experiments (Figures 5A,B) suggest that the level of Aor does not vary between the two conditions PS2 and ES20 and that the expression of the gene is even higher on ES20 than PS2 in stationary phase, contrary to what is observed for Adh. The amount of Aor is the same for the WT strain as for the complementation strain on ES20 (Figure 5A). These results suggest that different transcriptional regulators regulate the expression of the *adh* and *aor* genes, *adh* being more strongly expressed on PS2 while *aor* is more strongly expressed on ES20 leading to a similar amount of Aor enzyme on both media.

### 3.5. Growth analysis of the ADH-CG1 (Δ*adh*) mutant in pyruvate and ethanol media

**Figure 6:**
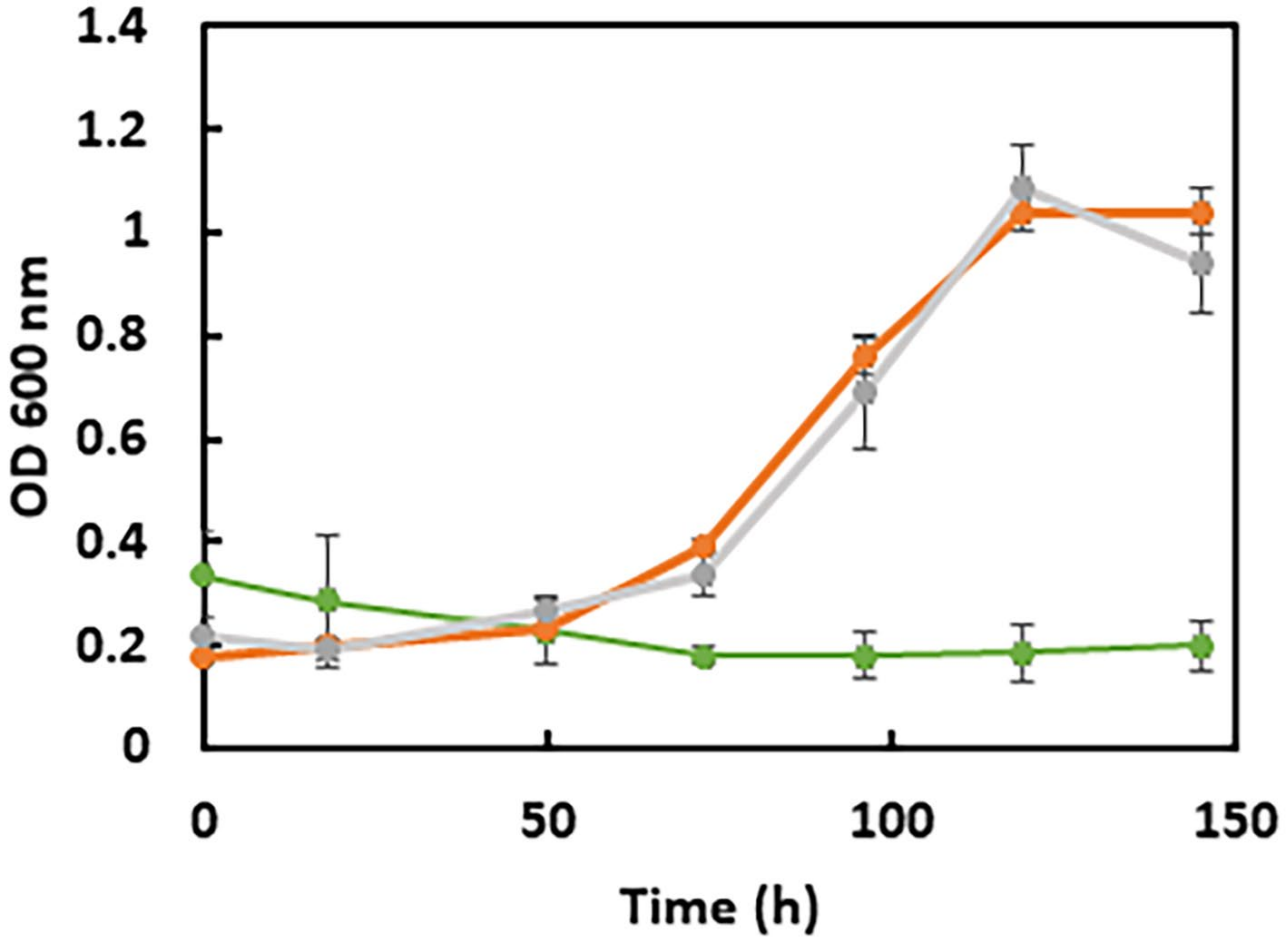
Growth curves of *S. fructosivorans* ADH-CG1 strain deleted of the Adh3929 encoding gene. Bacteria were grown in 100 mL serum bottles containing 90 mL of PS2 (orange curve), PS20 (gray curve) and ES20 (green curve) media. Data represent the averages of the results of three replicate growths. Error bars correspond to standard deviations.

We constructed the Adh-encoding gene deletion mutant, called the ADH-CG1 strain, and compared its growth to the WT strain (Figure 6). On pyruvate media, the ADH-CG1 strain grows well despite the fact that the generation time is higher than for the WT strain (30.9 ± 0.6 h for the mutant vs. 17.2 ± 0.9 h for the WT strain in PS2 medium and 28.0 ± 5.3 h for the mutant vs. 20.5 ± 0.3 h for the WT strain in PS20 medium) (Payne et al., 2022). On both media, the growth yield (OD_600_ max) is similar and is around 1 while it is slightly higher for the WT strain (between 1.2 and 1.4) (Payne et al., 2022). In contrast, on ES20 medium, the Δ*adh* mutant did not grow, indicating that Adh3929 is responsible for ethanol oxidation and that this enzyme is essential for bacterial growth under these conditions.

## 4. Discussion

In this work, we investigated the role of the electron-bifurcating Hnd hydrogenase in the physiology of *S. fructosivorans* by comparing the WT strain and SM4, the Hnd hydrogenase deletion mutant when the bacteria use ethanol as a carbon source. When Ollivier and colleagues characterized the *S. fructosivorans* species, they determined that it is able to oxidize ethanol in the presence of sulfate, much like related species from the *Desulfovibrio* genus (Ollivier et al., 1988). In low sulfate medium, the growth of *Desulfovibrio* is slight, and little of the ethanol is oxidized unless bacteria are co-cultured with an H_2_-consuming organism (Bryant et al., 1977). This syntrophic association between two partners, a H_2_-producing and a H_2_-scavenging partner is particularly important in anaerobic environments. Moreover, the electron transfer could even take place between the two microorganisms of the co-culture, not only by interspecies H_2_ transfer, but also directly as recently proposed by Zheng and colleagues, allowing the growth of *Desulfovibrio* in co-culture on ethanol in absence of sulfate (Zheng et al., 2021). As expected, we observed, in this study, that the WT strain is able to grow on a medium containing ethanol and sulfate but not on a medium containing ethanol only, while the growth of the SM4 mutant strain is completely prevented on both media, which confirms the involvement of Hnd in ethanol metabolism. Results obtained on the complementation strain indicate that contrary to what is observed on pyruvate media where the complementation is only partial in respiration (PS20) or in fermentation (PS2) (Payne et al., 2022), the presence of the *hnd* operon on the plasmid in the complementation strain restores the growth of the strain. These results also indicate that the amounts of the three enzymes Hnd, Adh and Aor are comparable in the two strains WT and SM4/pBGhnd6 and that the phenotype observed for the SM4 mutant strain is due to the *hndD* deletion. We have indeed shown previously that Hnd consumes, in a non-essential role, part of the H_2_ produced during pyruvate fermentation and produces reducing equivalents in the form of NADH and reduced ferredoxin through the electron bifurcation mechanism. These reducing equivalents are used for ethanol production as a fermentative product (Payne et al., 2022, 2023). In this study, our results show that Hnd is essential for the growth of *S. fructosivorans* on ethanol, indicating the importance of its role and highlighting for the first time an essential role of a hydrogenase for the growth of a *Desulfovibrio*. Previous studies of *Desulfovibrio* hydrogenase deletion mutants have never demonstrated the essential character of a hydrogenase in these bacteria (Rousset et al., 1991; Malki et al., 1997; Casalot et al., 2002a,b; Haveman et al., 2003; Caffrey et al., 2007; Morais-Silva et al., 2013). Moreover, it is important to note that none of the five other hydrogenases of *S. fructosivorans* compensates for the loss of Hnd, not even Hnt, the trimeric FeFe hydrogenase whose genes were identified in the genome (Baffert et al., 2019) and whose structure is very similar to the trimeric FeFe flavin-based electron-bifurcating hydrogenase from *Thermotoga maritima* (Schut and Adams, 2009). This putative trimeric hydrogenase Hnt has never been purified and biochemically characterized in any *Desulfovibrio* species and no evidence concerning its possible physiological role is available.

**Figure 7:**
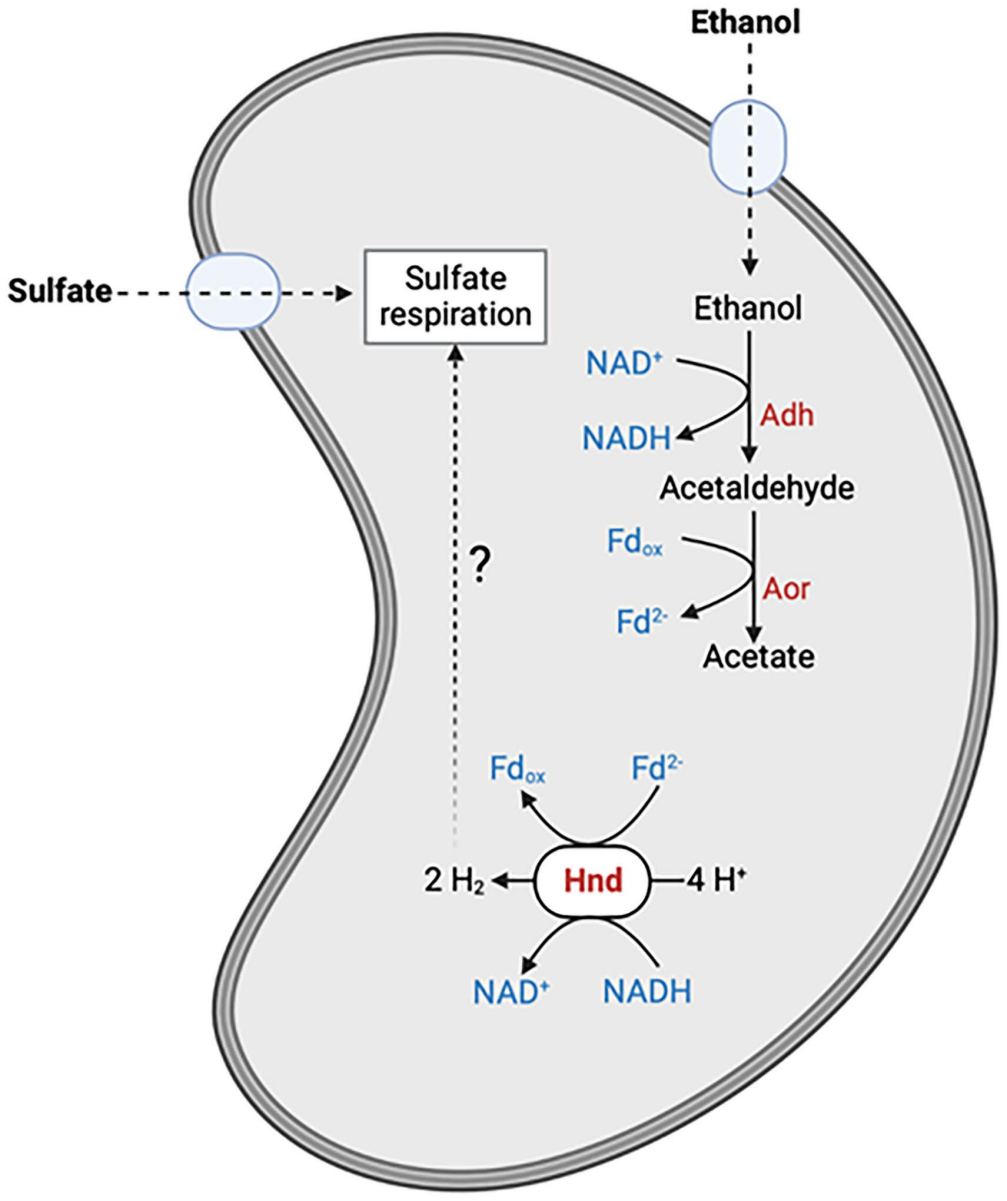
Model of the ethanol oxidation pathway in *S. fructosivorans*. Hnd hydrogenase is involved in the re-oxidation of NADH and reduced ferredoxin which are produced during ethanol oxidation by the enzymes Adh and Aor, through an electron confurcation mechanism. This leads to the production of H_2_ which could be recycled for sulfate reduction. The question mark indicates that this electron transfer pathway is hypothetical. Adh, alcohol dehydrogenase; Aor, Aldehyde ferredoxin oxidoreductase; Fd_ox_, oxidized ferredoxin; Fd^2−^, reduced ferredoxin. This figure was created with BioRender.com.

Furthermore, although the WT strain does not grow in ES0, it survives and accumulates H_2_ in the headspace whereas the SM4 mutant does not (Figure 2). A very small amount of ethanol must be consumed under these conditions to sustain this low H_2_ production but it is probably too low to be detectable on Figure 2A and to allow the growth of the bacteria. Our results led us to propose that Hnd produces H_2_ by an electron confurcation mechanism and regenerates the reducing equivalents, NADH and reduced ferredoxin, produced during ethanol oxidation by the two enzymes Adh which converts ethanol to acetaldehyde, and Aor which converts acetaldehyde to acetate (Figure 7). The H_2_ produced could be used for cytoplasmic sulfate reduction, probably by the mechanism for energy coupling of H_2_ cycling (Odom and Peck, 1981). Hnd is thus a reversible electron bifurcating hydrogenase that operates in H_2_ consumption during pyruvate fermentation and in H_2_ production when the bacterium uses ethanol as electron donor. Surprisingly, the expression of *hndD* in WT cells is much lower under pyruvate fermentation conditions than under ethanol oxidation conditions, which results in a lower amount of Hnd, although it is essential in the latter condition (Figure 3). The same is true for Adh3929 (Figure 4). We demonstrated that Hnd is able to catalyze H_2_ production using electron confurcation *in vitro*. The specific activity measured is low compared to the electron bifurcation activity. This is probably due to the low specific activity (and the limited quantity that can be added in the assay) of the PFOR used to generate reduced ferredoxin, that limits the overall confurcation activity. Moreover, we cannot directly compare the specific activities of electron confurcation vs. bifurcation because the driving force of the reactions is not the same and cannot be adjusted; it depends on the proportion of oxidized and reduced forms of the two electron donors/acceptors [on the Fdx(oxidized)/Fdx(reduced) ratio and on the NAD^+^/NADH ratio]. These ratios are also unknown in bacterial cells. Although it is not possible to discuss the variation in Hnd amount in the light of confurcation/bifurcation activity *in vivo*, our results demonstrate that Hnd is a bidirectional hydrogenase.

Only a few bacterial electron-bifurcating FeFe hydrogenases have been purified and characterized and the role of some of them has been determined. In acetogenic bacteria, such as *Acetobacterium woodii* and *Moorella thermoacetica*, the electron-bifurcating hydrogenase is involved in H_2_ consumption and produces NADH and reduced ferredoxin which are used for CO_2_ reduction (Schuchmann and Müller, 2012; Wang et al., 2013). In glucose-fermenting bacteria, such as *Ruminococcus albus* and *T. maritima*, the trimeric hydrogenase is involved in the production of H_2_ from NADH and reduced ferredoxin produced during fermentation through an electron confurcation mechanism to dissipate excess reducing power (Schut and Adams, 2009; Zheng et al., 2014; Greening et al., 2019).

The results obtained in this study and in our previous study clearly demonstrate that the *hnd* operon expression is strongly regulated at the transcriptional level. We have predicted the presence of a Rex-binding site in the promoter region of *hnd* and proposed that the expression of Hnd might be regulated by Rex, a transcriptional redox-sensing regulator controlled by the intracellular NAD^+^/NADH ratio. Moreover, HydS, a putative H_2_-sensing hydrogenase, whose mechanism has yet to be elucidated, might also be involved in the regulation of Hnd (Payne et al., 2022). When *R. albus*, a rumen bacterium, grows in co-culture with a hydrogenotrophic microorganism keeping the H_2_ partial pressure very low, the trimeric electron-bifurcating hydrogenase is expressed and produces H_2_. Its expression is tightly regulated, as is the expression of the two other hydrogenases of the bacterium, in response to H_2_ partial pressure. It has been proposed that the Rex regulator as well as the HydS hydrogenase are involved in the regulation of the expression of this hydrogenase (Zheng et al., 2014; Greening et al., 2019).

The expression of *adh* is also regulated at the transcriptional level and surprisingly, under respiratory condition, it is not induced by ethanol but is dependent on *hnd.* Indeed, while the WT strain grows well on ES20 ethanol medium (Figure 1), the SM4 strain shows completely impaired growth suggesting that, as is the case on pyruvate (Payne et al., 2022), the deletion of *hndD* induces a drastic decrease in the expression of *adh* on ethanol medium, making it impossible for the deletion mutant to use ethanol. However, the mechanism of regulation involved remains to be elucidated. In DvH, the mutant strain deleted of the periplasmic FeFe-hydrogenase shows also a very drastic drop in the expression of the *adh* gene, whereas it is one of the most expressed in the WT strain. Moreover, *adh* is upregulated when DvH uses ethanol as electron donor instead of lactate, pyruvate, formate or H_2_ indicating that the expression, unlike in *S. fructosivorans*, is induced by ethanol (Haveman et al., 2003). In this study, we also constructed the ADH-CG1 mutant strain deleted of the gene encoding Adh3929. The fact that this mutant strain does not grow on ethanol shows that Adh3929 is responsible for the oxidation of ethanol on ES20 and that this enzyme is essential for bacterial growth under these conditions. The counterpart of this Adh in DvH has also been shown to be responsible for the utilization of ethanol as the sole source of carbon and energy (Haveman et al., 2003). It is of note that although in the *S. fructosivorans* genome, 7 genes are annotated as “iron-containing alcohol dehydrogenase” in addition to the *adh*3929 gene (Supplementary Table S1), none of them compensates for the strong decrease of *adh*3929 expression in the *hndD* deletion mutant or for the lack of Adh3929 in the ADH-CG1 mutant to allow bacterial growth. This suggests that these *adh* genes are tightly regulated or that they code for alcohol dehydrogenases that do not use ethanol but other alcohols as a substrate. Interestingly, one of them, Adh1011, shows very strong homology to Adh 3929 (85% of protein sequence identity). However, the role of the other *adh* genes and the substrate specificity of the corresponding enzymes remain to be determined. Regarding *aor*, its expression profile is different compared to *hnd* and *adh* under ethanol condition while on pyruvate, the three genes followed the same profile (Payne et al., 2022). *aor* is over-expressed under ethanol condition as compared to pyruvate fermentation leading to a similar abundance of the enzyme under both conditions (Figure 5). These results again suggest that *aor* is regulated at the transcriptional level and that ethanol may be involved in the regulation of its expression. For the three operon/genes *hnd*, *adh*, and *aor*, the mechanism of regulation of their expression remains to be elucidated. Surprisingly, ethanol is not involved in the regulation of *hnd* and *adh* expression, because their expression is not upregulated under these conditions.

In DvH, the enzyme complex Flx-Hdr (Flx for flavin oxidoreductase and Hdr for heterodisulfide reductase), proposed to also perform electron bifurcation, plays a role that strikingly resembles the role of the hydrogenase Hnd (Ramos et al., 2015). This soluble complex is composed of six subunits, the 3 Flx subunits are homologous to subunits of *Pyrococcus* soluble hydrogenases but without a hydrogenase catalytic subunit and the 3 Hdr subunits sharing sequence homology with the archaeal HdrABC enzyme. In methanogenic archaea, the HdrABC enzyme forms a complex with the MvhADG NiFe-hydrogenase that catalyzes heterodisulfide CoM-S-S-CoB reduction with H_2_ coupled to the reduction of ferredoxin by flavin-based electron bifurcation (Thauer et al., 2008; Kaster et al., 2011; Wagner et al., 2017). The Flx-Hdr complex has been shown to be essential for ethanol oxidation in DvH by coupling the reduction of a ferredoxin and a disulfide (the DsrC protein) by NADH. In pyruvate fermentation, the Flx-Hdr complex is involved in ethanol production in a non-essential way by operating in reverse to reduce NAD^+^. Contrary to what we have shown in *S. fructosivorans* for the *hnd* operon, the *flx-hdr* genes are increased in transcription during growth in ethanol-sulfate in DvH. DvH does not possess an electron bifurcating hydrogenase, so it is the Flx-Hdr complex that plays the same essential role as Hnd in the oxidation of ethanol in this bacterium. On the other hand, the genome of *S. fructosivorans* contains a cluster with seven genes sharing sequence homology with genes encoding the Flx-Hdr complex of DvH (locus tags DesfrDRAFT_3930–3936) but the role of this complex is still unknown in *S. fructosivorans*. It is noteworthy that the *flx-hdr* gene cluster in *S. fructosivorans* is located immediately downstream of the *adh*3929 gene, as is the case in DvH (Ramos et al., 2015), which could also indicate a role in ethanol metabolism in this bacterium. However, Flx-Hdr does not take over ethanol oxidation when the hydrogenase Hnd is deleted. Our results as well as the results of Ramos and colleagues (Ramos et al., 2015) demonstrating the essential role of Hnd in *S. fructosivorans* as well as of the Flx-Hdr complex in DvH for ethanol oxidation highlight the importance of electron bifurcation in the energy metabolism in *Desulfovibrio*.

In summary, the results obtained here on *S. fructosivorans* have provided conclusive evidence that the hydrogenase Hnd is essential for the growth on ethanol by oxidizing the reducing equivalents formed during ethanol oxidation and coupling this oxidation with H_2_ production using an electron confurcation mechanism. Hnd is a reversible hydrogenase that operates in electron-bifurcating or -confurcating mechanism depending on the growth conditions.

## Data availability statement

The raw data supporting the conclusions of this article will be made available by the authors, without undue reservation.

## Author contributions

MB and AK involved in conception, design of the study, and writing of the manuscript. AK, CG, NP, JR, MKB, RL, CB, LS, and MB involved in acquisition, analysis, and interpretation of the data. All authors reviewed the manuscript and approved the submitted version.

## Funding

This work was supported by the CNRS and Aix Marseille Université and the Région Sud (CR PACA – AAP Recherche 2020 volet général, projet No-O2). This project has received funding from the European Union’s Horizon 2020 Research and Innovation Program under the Marie Skłodowska-Curie (grant agreement no. 713750). Also it has been carried out with the financial support of the Regional Council of Provence-Alpes-Côtes d’Azur and with the financial support of the A*MIDEX (no. ANR-11-IDEX-0001-02), funded by the Investissements d’Avenir project funded by the French Government, managed by the French National Research Agency (ANR).

## Conflict of interest

The authors declare that the research was conducted in the absence of any commercial or financial relationships that could be construed as a potential conflict of interest.

## Publisher’s note

All claims expressed in this article are solely those of the authors and do not necessarily represent those of their affiliated organizations, or those of the publisher, the editors and the reviewers. Any product that may be evaluated in this article, or claim that may be made by its manufacturer, is not guaranteed or endorsed by the publisher.
