# Supplementary material for: An essential role of the reversible electron-bifurcating hydrogenase Hnd for ethanol oxidation in Solidesulfovibrio fructosivorans
Source: Front Microbiol. 2023 Mar 27;14:1139276. doi: 10.3389/fmicb.2023.1139276 (PMC10084766; doi:10.3389/fmicb.2023.1139276)
Supplement: Supplementary file 1 [file Table_1.docx]

Table S1 : Genes annotated as “iron-containing alcohol dehydrogenase” in the *S. fructosivorans* genome. The number of amino acids, the calculated molecular weight and the protein sequence identity with the Adh 3929 are indicated.

| Locus tag | GenBank accession | Number of amino acids | Molecular weight (kDa) | Protein sequence identity with Adh3929 (%) |
| --- | --- | --- | --- | --- |
| **3929** | **EFL49342** | **393** | **41.9** | **100** |
| 0219 | EFL53171 | 393 | 41.4 | 41 |
| 0766 | EFL52660 | 380 | 40.9 | 36 |
| 0953 | EFL52464 | 385 | 38.8 | 32 |
| 1011 | EFL52191 | 393 | 41.7 | 85 |
| 2367 | EFL50995 | 401 | 41.9 | 23 |
| 2711 | EFL50596 | 383 | 39.3 | 35 |
| 4071 | EFL49189 | 384 | 40.4 | 35 |
