# Supplementary material for: An essential role of the reversible electron-bifurcating hydrogenase Hnd for ethanol oxidation in Solidesulfovibrio fructosivorans
Source: Front Microbiol. 2023 Mar 27;14:1139276. doi: 10.3389/fmicb.2023.1139276 (PMC10084766; doi:10.3389/fmicb.2023.1139276)
Supplement: Supplementary file 2 [file Data_Sheet_1.pdf]

Figure S1

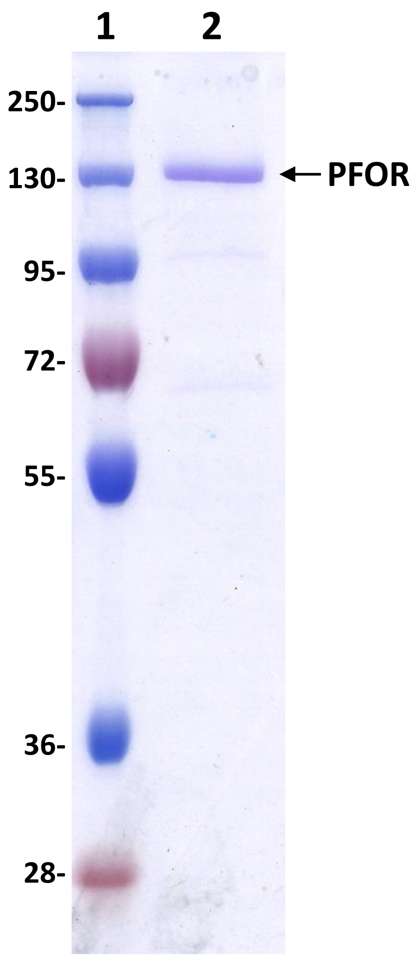

Figure S1: SDS-PAGE 10 % of the purified recombinant Strep-tagged PFOR from *S. fructosivorans* stained with Coomassie Blue. The identity of the enzyme was confirmed by mass spectrometry analysis. Lane 1: Molecular mass markers (in kDa); lane 2, purified PFOR.

Figure S2

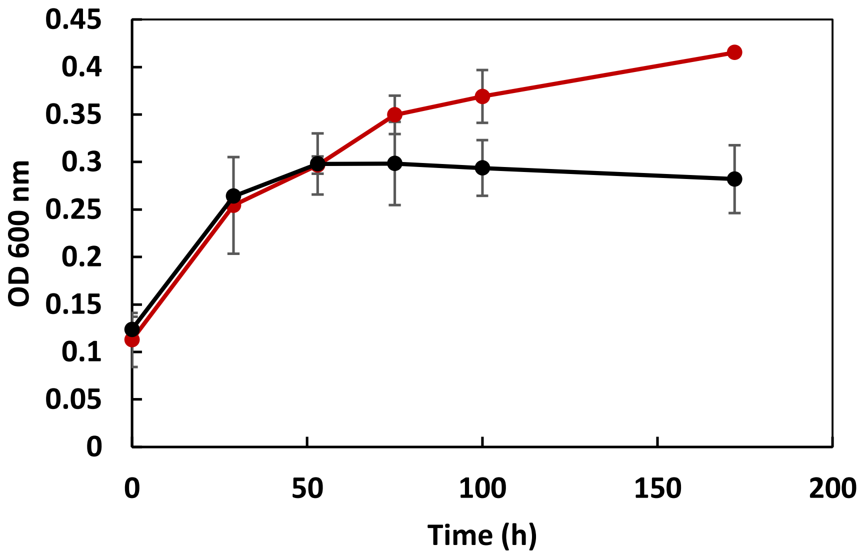

Figure S2: Growth curves of *Solidesulfovibrio fructosivorans* WT and SM4 strains. Bacteria were grown in 100 mL serum bottles containing 90 mL of H_2/sulfate medium. WT strain, black curve; SM4 strain, red curve. Data represent the averages of the results of three replicate growths. Error bars correspond to standard deviations.

Figure S3

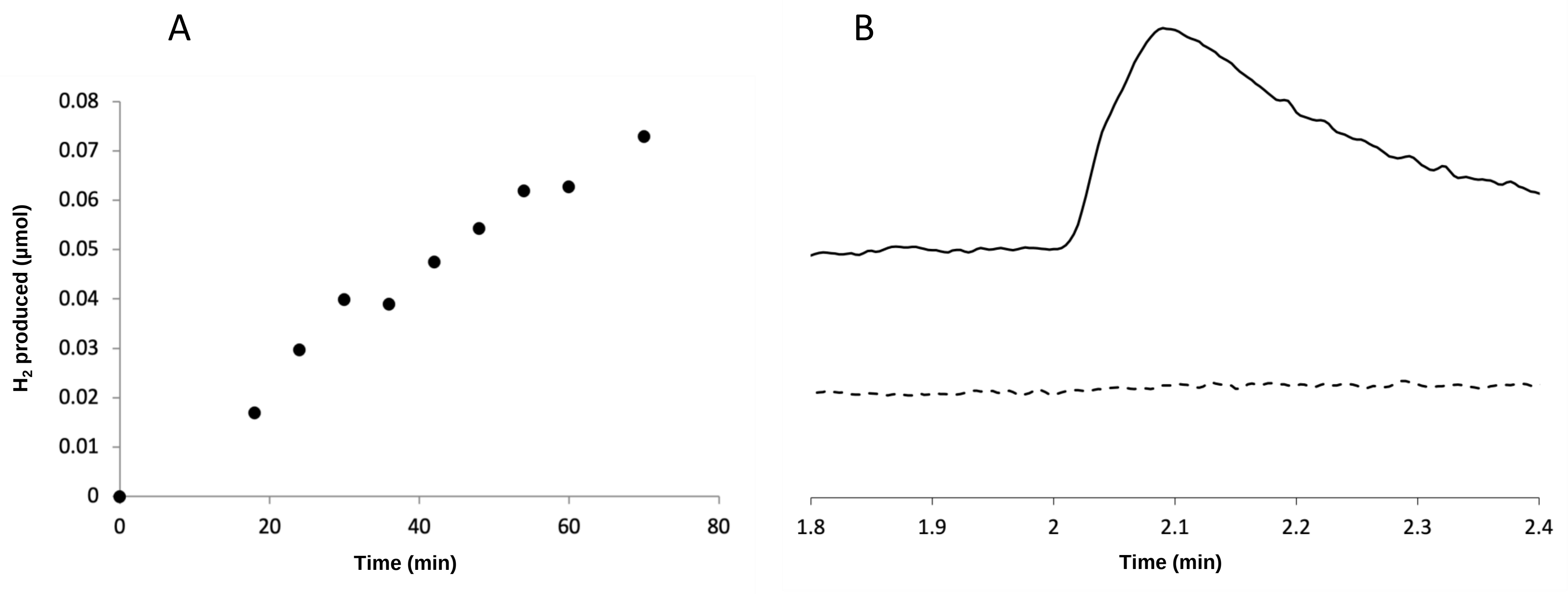

Figure S3: Electron confurcation assay of Hnd. (A) H_2 produced in the headspace during the assay. (B) Chromatogram at the start (t=0 min) of the confurcation assay (dotted line) and at t= 70 min (plain line). These results are representative of several similar experiments.

Figure S4

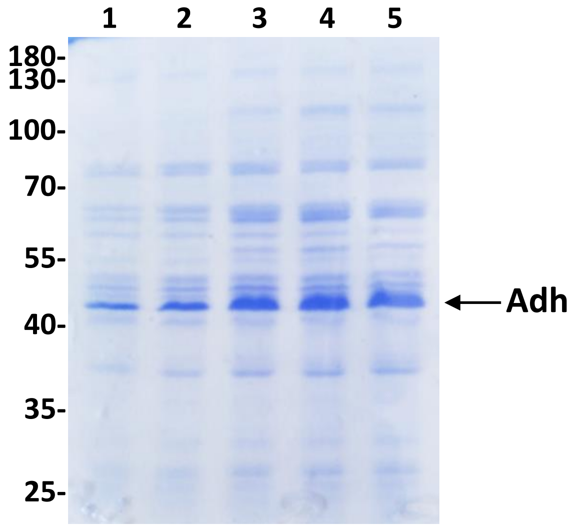

Figure S4: Coomassie blue-stained SDS-PAGE of total proteins from *S. fructosivorans* WT strain grown with pyruvate (PS2 medium). Cells were harvested during growth (lane 1, 25h; lane 2, 44h; lane 3, 67h; lane 4, 91h; lane 5, 116h). 5 µg of soluble proteins were loaded in each lane of a 12% SDS-PAGE. Molecular mass markers are indicated in kDa. Arrow indicates bands corresponding to Adh (3929). The theoretical molecular mass of Adh is 41.9 kDa.
